# Accuracy and dimensional reproducibility by model scanning, intraoral scanning, and CBCT imaging for digital implant dentistry

**DOI:** 10.1186/s40729-021-00343-w

**Published:** 2021-06-30

**Authors:** Akira Komuro, Yoichi Yamada, Satoshi Uesugi, Hiroaki Terashima, Masashi Kimura, Hiroto Kishimoto, Tsutomu Iida, Katsuya Sakamoto, Kenichi Okuda, Kaoru Kusano, Shunsuke Baba, Takashi Sakamoto

**Affiliations:** 1 Osaka Academy of Oral Implantology, 1-1-43 Abenosuji, Abenoku, Osaka, 545-6008 Japan; 2 Department of Oral Implantology, Osaka Dental University, 1-5-17 Otemae Chuo-ku, Osaka, 540-0008 Japan

**Keywords:** Digital implant dentistry, Surgical guide, Model scanner, Intraoral scanner, CBCT

## Abstract

**Background:**

Recently, it has become possible to analyze implant placement position using the digital matching data of optical impression data of the oral cavity or plaster models with cone beam computed tomography (CBCT) data, and create a highly accurate surgical guide. It has been reported that CBCT measurements were smaller than the actual values, termed shrinkage. Matching of digital data is reliable when the plaster model or intraoral impression values show shrinkage at the same rate as the CBCT data. However, if the shrinkage rate is significantly different, the obtained digital data become unreliable. To clarify digital matching reliability, we examined dimensional reproducibility and shrinkage in measurements obtained with a model scanner, intra-oral scanner (iOS), and CBCT.

**Materials and methods:**

Three implants that were arranged in a triangle were fixed in an acrylic plate. The distance between each implants were measured using model scanner, iOS, and CBCT. The actual size measured by electronic caliper was regarded as control.

**Results:**

All values measured with CBCT were significantly smaller than that of model scanner, iOS, and control (*p<*0.001). The model scanner shrinkage was 0.37-0.39%, iOS shrinkage was 0.9-1.4%, and CBCT shrinkage was 1.8-6.9%. There were statistically significant differences among the shrinkage with iOS, CBCT, and model scanner (*p<*0.001).

**Conclusion:**

Our findings showed that all measurements obtained with those modalities showed shrinkage as compared to the actual values. In addition, CBCT shrinkage was largest among three different measuring methods. They indicated that data matching between CBCT and scanner measurements requires attention in regard to the reliability of values obtained with those devices.

## Introduction

In recent years, the so called top-down treatments have been widely used to simulate implant placement using findings obtained with cone beam computed tomography (CBCT) by taking into account the design of the superstructure [1, 2]. Three-dimensional radiographic imaging using CBCT was first introduced in 1998 and has become an established diagnostic technique in various dental fields including implant dentistry [3, 4]. Compared with traditional radiographic methods, CBCT offers volumetric data on jaw bones and teeth [5]. Initially, conventional multi-slice computed tomography was used; dental cone beam computed tomography rapidly became more popular due to relatively low radiation doses and reasonable costs [6]. Therefore, CBCT has been widely used as a powerful tool in implant diagnosis and surgical planning. Furthermore, it has become possible to perform accurate implant placement by digital matching of optical impression data of the oral cavity or plaster models with CBCT data. It has become possible to analyze implant placement position using the digital matching data and create a highly accurate surgical guide [6, 7]. However, our previous study showed that CBCT measurements were smaller as compared to the actual values, termed shrinkage [8].

Matching of digital data is reliable when the plaster model or intraoral impression values show shrinkage at the same rate as the CBCT data. However, if the shrinkage rate is significantly different, the obtained digital data become unreliable. Moreover, model scanners are used more frequently than intra-oral scanners (iOS) in clinical practice and have additional factors in regard to dimensional changes, such as expansion with hardening, making it difficult to confirm whether appropriate digital matching has been obtained. Even though there are factors such as shrinkage of the data and dimensional deformation of the resin when making the surgical guide from the matching data, in actual clinical practice, the surgical guide can be set in the mouth with slight adjustments. This is because the degree of shrinkage is not so great as to make it impossible to set, and because of the elasticity of the resin. However, in the severe cases of implant placement, it is important to consider the fact that the data shows shrinkage. In order to clarify the reliability of digital matching, we examined dimensional reproducibility and shrinkage in measurements obtained with model scanner, iOS, and CBCT examinations performed using the same subject model.

## Materials and methods

**Fig. 1:**
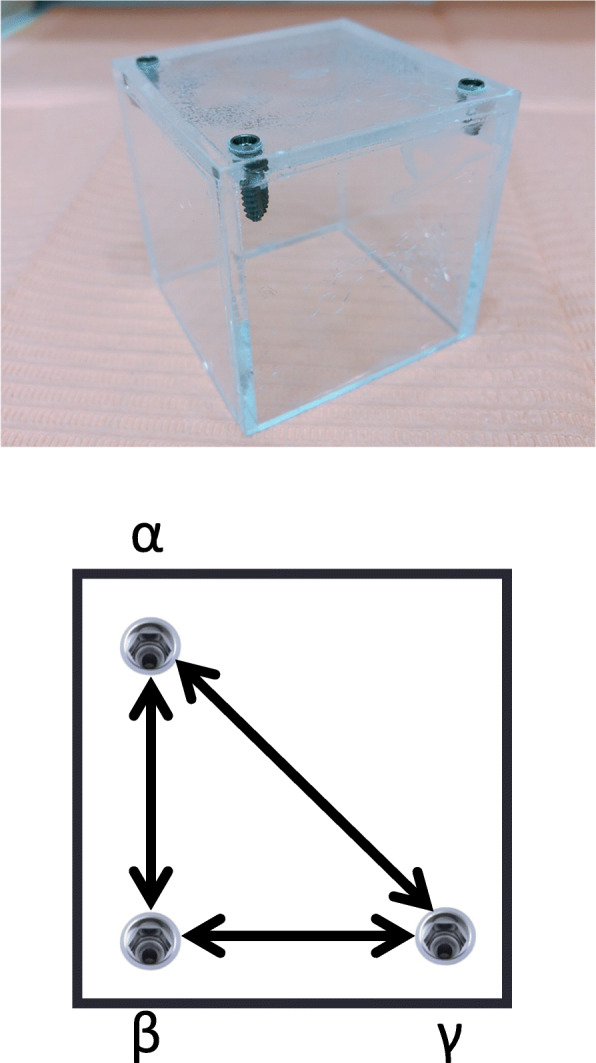
Model used for scanning. Three titanium screw type implants were fixed to an acrylic plate and arranged in a triangle, with the side lengths ranging from 4-5 cm and designated as *α*, *β*, and *γ*

Three titanium screw type implants (Xive, Dentsply Sirona, USA), each measuring 3.4 mm in diameter and 11 mm in length, were fixed in an acrylic plate. They were arranged in a triangle with each side approximately 4-5 cm in length, and designated as *α*, *β*, and *γ* (Fig. 1). The implants distance was set concerning the average distance between the left and right first molars and between the first molars and adult incisors. For the CBCT examination, a Veraviewepocs 3Df device (Morita, Kyoto, Japan) (voxel size: 125 μm, FoV: Ø100 × H50/80) was used. Cerec Omnicam (Dentsply Sirona, USA) was used for iOS. For the model scanning, the model was constructed of high-strength dental stone (Newfujirock, GC, Tokyo, Japan) after application of a silicone rubber impression material (Dent Silicone Aqua, Shofu, Kyoto, Japan). Then, a Cerec InLabo (Dentsply Sirona, USA) was used for scanning. After CBCT imaging, model scanning and intra-oral scanning, the distances between each implant were automatically visualized by the included software and measured 10 times by 3 dentists with more than 20 years of general clinical experience, with the final values agreed on by consensus. The imaging conditions for CBCT were 90 kV at 5 mA. Actual dimensions between the implants were defined as control. They are determined by measuring 10 times for each implant using an electronic caliper (IP67, HoLEX, Germany).

The statistical analyses were performed using the SigmaPlot software 12.3. Differences among the experimental groups were examined by one-way analysis of variance (ANOVA) tests using Tukey’s honest significant difference test. A *p* value less than 0.05 was considered to be statistically significant.

## Results

**Fig. 2:**
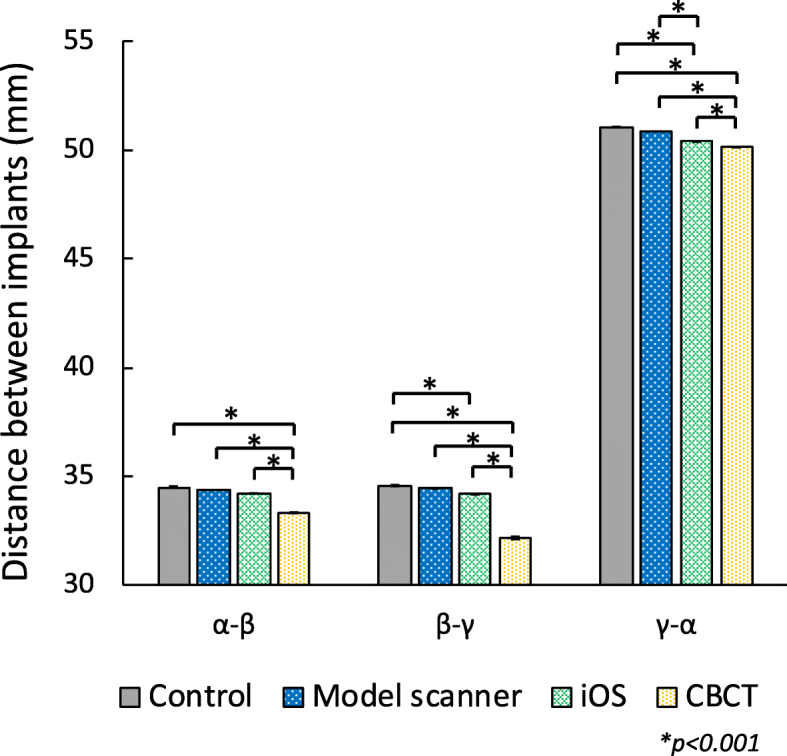
Distance between each implant. The vertical axis shows the measurements obtained by the model scanner, iOS, and CBCT. Bar: standard deviation

The measurements between each implant are shown in Fig. 2. Mean values for the actual measured size (control) and that obtained by the model scanner, iOS, and CBCT for the distance between *α* and *β* (α - β) were 34.5, 34.37, 34.22, and 33.33 mm, respectively, between *β* and *γ* (β - γ) were 34.57, 34.44, 34.16, and 32.17 mm, respectively, and between *γ* and *α* (γ - α) were 51.06, 50.86, 50.39, and 50.13 mm, respectively. The measurements by CBCT were statistically significantly smaller than that of control, model scanner, and iOS at all measurement position (*p* < 0.001). Data obtained by iOS were also significantly smaller than control at *β - γ* and *γ - α* (*p <* 0.001). There were no significant differences between measurements by model scanner and control.

**Fig. 3:**
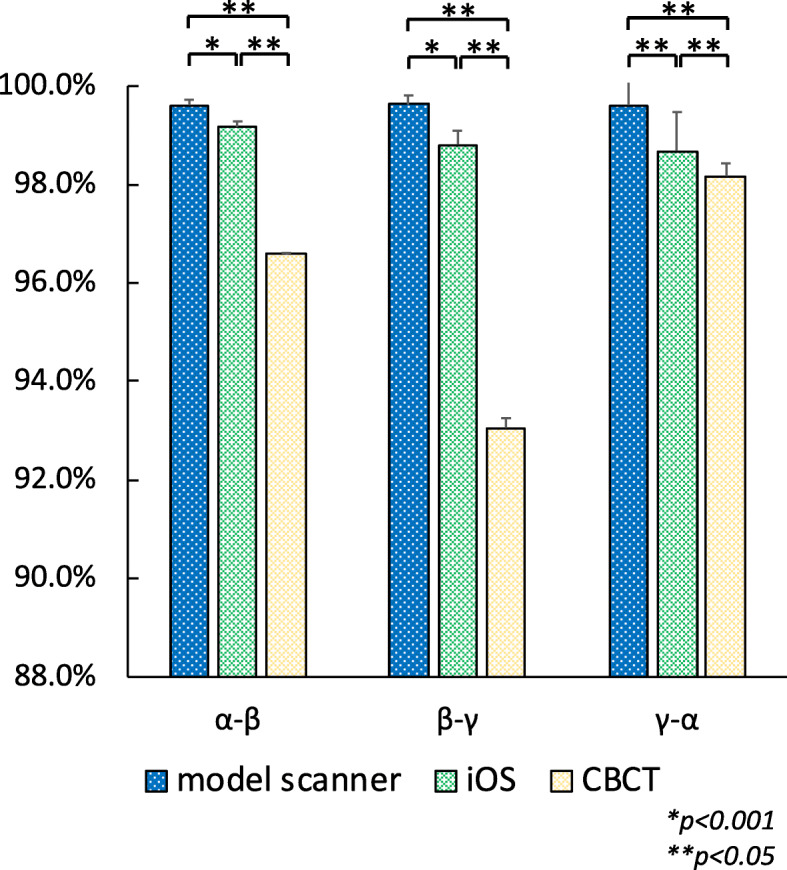
The percentage of measurements by the model scanner, iOS, and CBCT compared to the control. Bar: standard deviation

In order to clarify the differences among model scanner, iOS, and CBCT, the percentage of measurements by the model scanner, iOS, and CBCT compared to control were analyzed (Fig. 3). CBCT was statistically significantly smaller than both model scanner and iOS at all measurement position (*p <* 0.001). There were also significant differences between model scanner and iOS at *α - β* and *β* - *γ* (*p* < 0.05), and at *γ* - *α* (*p* < 0.001).

**Fig. 4:**
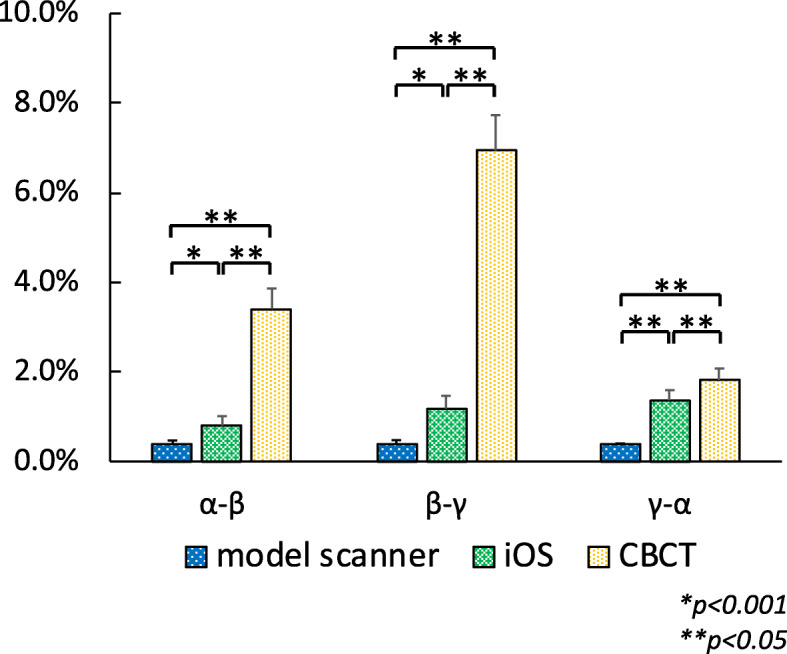
The shrinkage rate of the model scanner, iOS, and CBCT compared to control. Bar: standard deviation

The percentage of shrinkage of the model scanner, iOS, and CBCT was shown in Fig. 4. The average shrinkage rate of the model scanner, iOS, and CBCT for the distance of *α - β* were 0.37±0.1%, 0.81±0.2%, and 3.41±0.45%, respectively, between *β* and *γ* were 0.37±0.11%, 1.19±0.28%, and 6.94±0.79%, respectively, and between *γ* and α were 0.38±0.02%, 1.37±0.22%, 1.83±0.26%, respectively. The shrinkage rate of CBCT was statistically significantly higher than that of model scanner and iOS (*p <* 0.001). There were also significant differences in the shrinkage rate between model scanner and iOS at *α - β* and *β* - *γ* (*p <* 0.05), and at *γ - α* (*p <* 0.001).

## Discussion

In order to clarify the reliability of digital matching, we examined dimensional reproducibility and shrinkage in measurements obtained with model scanner, iOS, and CBCT examinations performed using the same subject model. All values measured with CBCT were significantly smaller than that of model scanner, iOS, and control (*p*<0.001). The model scanner shrinkage was 0.37-0.39%, iOS shrinkage was 0.9-1.4%, and CBCT shrinkage was 1.8-6.9%.

Since the introduction of digital models in 1990, several ways were developed such as CBCT, iOS, laser scanning, holographic scanning, and stereophotogrammetry [9]. The use of such different types of digital models has increased due to their advantages over plaster models. Especially, CBCT has been used increasingly, because CBCT can obtain more information such as bone levels and root positions [9]. In addition, CBCT is possible to perform virtual setups to simulate results of implant treatment. However, it is still unclear whether the use of software to perform measurements and calculations facilitates and accelerates the process of dental analysis.

**Fig. 5:**
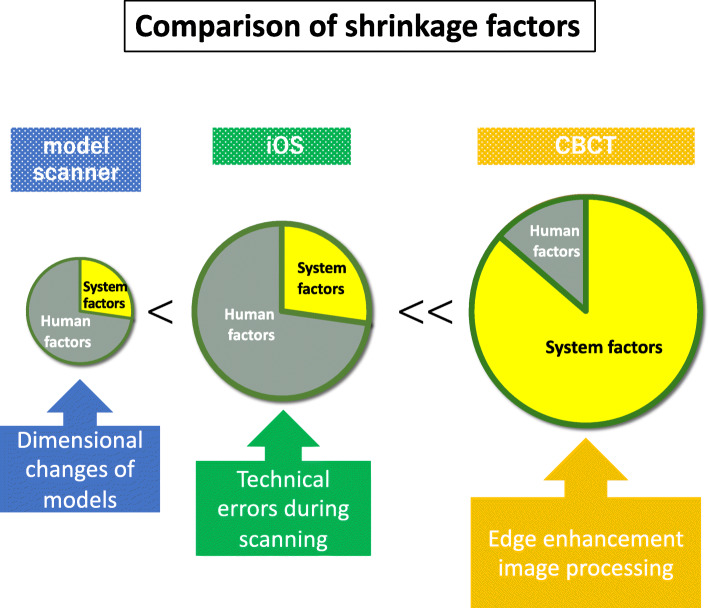
Factors related to CBCT and scanner measurement shrinkage

Factors related to shrinkage in measurements obtained by CBCT as compared to the actual values include those related to both hardware and software, as well as human error (Table 1). As for factors related to hardware, it is important to note that the amount of correction is increased because the length of the X-ray tube detector in a panorama multifunction machine is short and the opening angle of the cone beam is wide, while factors related to software are caused by the processes used for edge enhancement and metal artifact reduction. As for human error, that is considered to be primarily related to the individual performing the measurements. In the present study, all measured values were smaller than the actual size because of these factors causing such shrinkage. Factors causing shrinkage of measurements obtained with the model scanners and iOS were also considered to be related to hardware, software, and human error (Table 1). Hardware issues are caused by the performance and scanning mode of the camera body [10], while software issues are related to the CAD software package, and human error is caused by technical errors by the operator during scanning as well as plaster cast production. Shrinkage was smallest with the model scanner, followed by the iOS, and CBCT (Fig. 5). This is thought to be caused that model scanner has larger range for scanning at one time.

**Table 1:** Overview of shrinkage factors by model scanner, iOS, and CBCT

	Model scanner	iOS	CBCT
**System factors**			
Hardware factors	Camera body	Camera body	CBCT
Software factors	CAD software	CAD software	Processing software
**Human factors**	Dimensional changes of models	Technical errors during scanning	Measurement errors
**Amount of shrinkage**	+	++	+++

In order to consider human and non-human factors separately, hardware and software issues were defined as system factors, then further analysis was conducted. With CBCT, inaccurate measurements caused by human error are very limited, and most of the shrinkage was considered to be caused by system factors. Clinical studies have shown that various imaging objects in the mouth, such as teeth, bones, soft tissues, and metal prostheses, have complex effects on imaging and data processing [11, 12]. The iOS accuracy is also affected by the existence of objects in the mouth, though recent advancements in hardware and software design have been remarkable; thus, it is considered that the influence of human factors is rather large. In other words, the scanning technique of the operator is greatly related to accuracy, including basic techniques such as handling of the camera in cases with a metal prosthesis and moisture condition. With model scanner measurements, though the presence of a metal prosthesis has no influence, dimensional changes of plaster models, such as hardening that causes expansion, are considered to be the major factors leading to errors.

Based on the background information noted above and our findings, the following points are considered important to reduce data matching errors. When an intra-oral scanner is employed, mastery of the scanning method is necessary. Furthermore, variations in the ratio of expansion of the plaster model due to hardening can be suppressed by use of an appropriate water ratio for mixing. With CBCT, use of that modality for patients with few metal artifacts caused by metal prostheses is important to reduce system error. Regardless of the method used, the following points are crucial. First, attention must be given to selection of a reference point for data matching. Also, data discrepancies increase with an increased number of implants [13]. Finally, verification of matching accuracy is required for each combination of factors when using CBCT, scanners, and processing software, because CBCT devices use different data contraction modes depending on the manufacturer [14], while model and intra-oral scanners show various levels of accuracy that are dependent on scan mode and recommended scanning method.

Compared to in vitro setups, in a clinical settings, we are likely to be more affected by the “human factors.” The difference in the data will be larger in clinical settings and will affect the proper planning and creation of the surgical guide. Therefore, in the severe cases of implant placement, it is important to take into account the fact that the data shows shrinkage.

## Conclusion

In the present study, we determined dimensional reproducibility and shrinkage ratio using model scanners, iOS, and CBCT with the same subject model to clarify the reliability of digitally obtained measurements. Our findings showed that all measurements obtained with those modalities showed shrinkage as compared to the actual values, with the average and variance greatly different among the 3 methods. In addition, they indicate that data matching between CBCT and scanner measurements, which showed great differences for both average and variance of shrinkage, requires attention in regard to the reliability of values obtained with those devices.

## Data Availability

The datasets used and analyzed during the current study are available from the corresponding author on reasonable request.
